# Comparison of Genomes of Species from *Polemonium caeruleum* Complex and *Polemonium pulcherrimum* Complex Based on Repeatome and Chromosome Analysis

**DOI:** 10.3390/ijms27010229

**Published:** 2025-12-25

**Authors:** Olga V. Muravenko, Alexandra V. Amosova, Alexey R. Semenov, Ekaterina D. Badaeva, Julia V. Kalnyuk, Svyatoslav A. Zoshchuk, Olga Yu. Yurkevich

**Affiliations:** Engelhardt Institute of Molecular Biology, Russian Academy of Sciences, 32 Vavilov St, Moscow 119991, Russia

**Keywords:** *Polemonium caeruleum* complex, *P. pulcherrimum* complex, genome, NGS, repeatome, satellite DNAs, 45S rDNA, 5S rDNA, FISH, chromosome variability

## Abstract

*Polemonium* L. (Polemoniaceae) is a widespread genus native to subarctic and arctic regions of the Northern Hemisphere. The taxonomy and genome relationships within *Polemonium* are still unclear. We analyzed genomes of three species from each *Polemonium caeruleum* and *Polemonium pulcherrimum* complex using bioinformatic analysis by RepeatExplorer2/TAREAN pipelines of next-generation sequencing data. The repeatomes of all studied species were similar in type and number of repeats. Satellite DNAs (satDNAs) demonstrated high sequence identity within the studied species. FISH chromosome mapping of 45S rDNA, 5S rDNA, and two satDNAs Pol_C 33 and Pol_C 46 allowed us to construct the species karyograms and assess the genome diversity within the *P. caeruleum* complex and *P. pulcherrimum* complex, and also confirm the taxonomic status of *P. kiushianum* as an independent species. Our findings demonstrate a close genomic relationship among the species from *P. caeruleum* and *P. pulcherrimum* complexes, indicating the presence of a common ancestral genome; additionally, our results provide cytogenetic evidence for the monophyletic origin of these sections and also complex evolutionary history of the genus *Polemonium*. The developed approach may be a valuable framework for further investigation of the chromosomal organization of karyotypes in other species of the genus *Polemonium*.

## 1. Introduction

The genus *Polemonium* is the most widespread in the Polemoniaceae family. It includes mainly perennial herbaceous plants, common in Eurasia and America [1,2,3]. Different scientific approaches, such as morphological and cytological analyses, yield estimates ranging from twenty-five to forty species within the genus *Polemonium* [2,3,4,5,6]. Most species are native to North America, with *P. micranthum* also found in southern South America. About fourteen species are distributed in Eurasia. However, the taxonomy and interspecific relationships of *Polemonium* species still remain controversial [2,3,4,7].

The system of the interspecies relationships within the genus *Polemonium* was first developed by Davidson based on morphological data [2]. Currently, taxonomists divide the species of *Polemonium* into four main species complexes [1,8,9]. The *P. caeruleum* complex (section *Polemonium*) includes species from Eurasia and partially from North America, with tall, erect plants native to wet meadows, marshes, and forest clearings. These taxa have elongated inflorescences and dark blue (sometimes white) bell-shaped corollas with yellow anthers [1,8]. The *P. pulcherrimum* complex includes dwarf taxa from Eurasia and the USA that grow in the alpine and subalpine zones. These taxa have corymbose inflorescences, blue bell-shaped corollas with white or yellowish tubes, and white anthers [1,8]. The *P. viscosum* complex include alpine taxa, growing primarily in the subalpine or subarctic biomes in the American Rocky Mountains, with crowded racemose inflorescences, funnel-shaped-tubular corollas of blue-violet or cream color, and bright yellow anthers [10]. Species of the *P. foliosissimum* complex are distributed only in the coniferous forests of the west-central USA. They are mid-elevation erect plants with corymbose inflorescences, coplanar leaflets, and yellow anthers [1,8].

High variability in morphological features complicates the identification of closely related species within the genus *Polemonium*. Intermediate forms are often found within and between complexes [1,2,3,11,12]. In regions where populations of the related *Polemonium* species grow together, the introgressive hybridization events might occur. Several species having the hybrid origin were previously described within *Polemonium* [3,12,13,14]. The boundaries between the species are blurred not only by interspecific hybridization but also by convergent evolution, and in some cases by both of these factors [4].

Elucidating the phylogenetic relationships within the genus *Polemonium* proved to be a rather complicated problem. However, Davidson [2] suggested the monophyly of the *Polemonium* species based on morphological characteristics. Grant [1] assumed that the *P. caeruleum* complex exhibited the most plesiomorphic states of the genus characters. According to molecular genetic studies, basal relationships between species complexes were not well resolved [8,15,16]. AFLP (amplified fragment length polymorphism) analysis showed a monophyletic origin of the genus *Polemonium* [8], which was later confirmed by the phylogenetic analysis based on nuclear DNA data [9]. However, the *Polemonium* genus was paraphyletic according to plastid data [9]. The discovery of strong plastid-nuclear mismatches in the studied species indicates the presence of introgression events (repeated acts of interspecific hybridization, as well as chromosome translocations and inversions) that could occur during speciation [9].

For species of the genus *Polemonium*, the basic chromosome number is *x* = 9, and most species are diploid (2*n* = 2*x* = 18) [5,6]. In the karyotypes of several *Polemonium* species (including *P. caeruleum* and *P. pulcherrimum*) similarity in chromosome morphology was detected [17]. Considering the presence of introgression events, including chromosomal rearrangements [9], molecular markers of chromosomes should be used for the comprehensive study of karyotypes of various *Polemonium* species.

In vascular plants, repetitive sequences such as satellite DNA (satDNA) and mobile elements are considered to be essential components of genomes and also driving forces of evolution [18,19]. SatDNA families are widely used as effective chromosomal markers to characterize plant genomes, assess intra- and interspecies genomic variability, and also in phylogenetic studies [20,21,22]. Recently, based on chromosome morphology and distribution patterns of the cytogenetic markers (rDNAs and satDNAs), the karyotype of *P. caeruleum* was studied and significant intraspecies chromosomal variability was revealed [23]. Moreover, it was suggested that the found effective markers could be used for the further analysis of interspecific genetic variability within the genus *Polemonium*.

In the present study, to detect intra- and interspecific genomic diversity among *Polemonium* species, a comparative bioinformatic analysis of repeatomes of *P. caeruleum* L. var. ‘Lazur’ (*P. caeruleum* complex) and *P. pulcherrimum* H., *P. boreale* Adams, and *P. villosissimum* (Hultén) D.F. Murray & Elven (=*P. boreale* var. *villosissimum* Hultén) (*P. pulcherrimum* complex) was conducted using the RepeatExplore2/TAREAN pipelines. Moreover, the karyotype structures of the species from the *P. caeruleum* complex (*P. caeruleum*, *P. kiushianum* Kitam., and *P. racemosum* (Regel) Kitam.) and *P. pulcherrimum* complex (*P. pulcherrimum* H. and *P. boreale* Adams) were examined using FISH-based chromosome mapping of the repetitive DNA probes, 45S rDNA, 5S rDNA, and two satDNAs.

## 2. Results

### 2.1. Repetitive DNA Identification Using the RepeatExplorer2/TAREAN/DANTE_LTR Pipelines

Using RepeatExplorer2/TAREAN pipelines, we carried out a comparative bioinformatic analysis of NGS genome data of the species belonging to two *Polemonium* complexes: *P. caeruleum* L. var. ‘Lazur’ (*P. caeruleum* complex) and also three species from *P. pulcherrimum* complex (*P. pulcherrimum, P. boreale*, and *P. villosissimum)*. The data for *P. caeruleum* were taken from our previous study [23].

**Figure 1:**
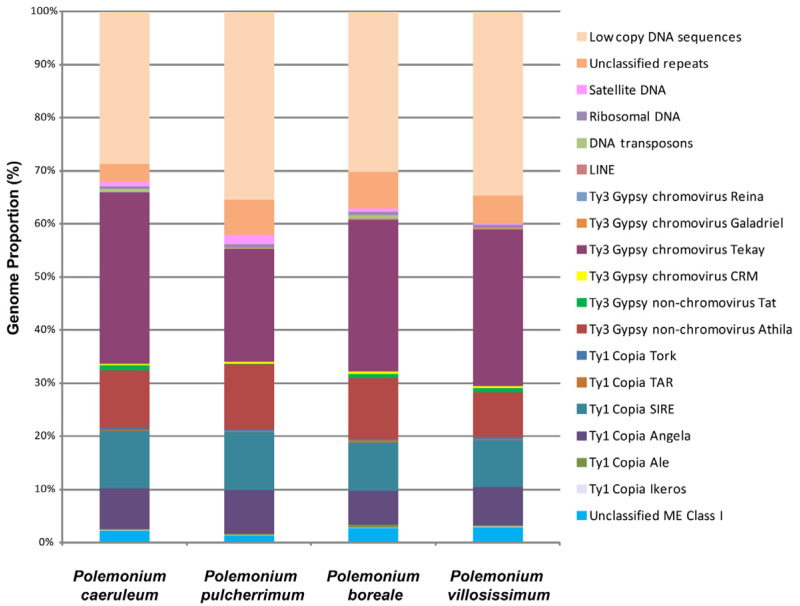
Types and genome proportions of the DNA repeats identified in the *Polemonium caeruleum*, *P. pulcherrimum, P. boreale*, and *P. villosissimum* genomes. The data for *P. caeruleum* were taken from our previous study [23]. Each proportion was calculated using RepeatExplorer2 as a ratio of the number of reads specific to a particular repeat type to the sum of all reads used in the cluster analysis.

According to the bioinformatic analysis, DNA repeats made up the majority of the genomes of the *Polemonium* species: 64.47% (*P. pulcherrimum*), 69.81% (*P. boreale*), 65.30% (*P. villosissimum*), and 71.25% (*P. caeruleum*) (Figure 1). Transposable elements (TEs) were most abundant in repeatomes of all studied *Polemonium* species (Supplementary Table S1, Figure 1). The highest number of TEs was found in the genome of *P. caeruleum*. The genome of *P. pulcherrimum* had the lowest number of TEs. The species *P. boreale* and a closely relative *P. villosissimum* had rather similar genome proportions of TEs.

Depending on the species, 55.33–66.08% of the detected TEs were retrotransposons (class I), and about 0.09–0.57% of TEs were DNA transposons (class II) (Supplementary Table S1, Figure 1). LTR retrotransposons were the most common class I mobile elements. They included 33.96–44.46% of the Ty3-Gypsy superfamily (mostly non-chromovirus Athila and chromovirus Tekay) and 16.85–19.91% of the Ty1-Copia superfamily (mostly SIRE and Angela). Among the studied species, Ty1-Copia elements were most abundant in *P. pulcherrimum*, and Ty3 Gypsy retroelements were most abundant in *P. caeruleum* (Supplementary Table S1, Figure 1).

The genomes of the studied species comprised 0.42–0.74% of ribosomal DNA. Satellite DNA constituted 0.17–1.64% of the studied genomes, and the highest amount of the satDNA was found in *P. pulcherrimum*. The genome of *P. villosissimum* contained less satDNA (0.17%) compared to its closely relative species *P. boreale* (0.68%) (Supplementary Table S1, Figure 1).

RepeatExplorer2/TAREAN pipelines identified 3–6 high-confidence putative satDNA families and 5–8 low-confidence putative satDNA families among the studied species of the *P. pulcherrimum* complex, and also 6 high-confidence putative satDNAs and 3 low-confidence putative satDNAs in the genome of the species *P. caeruleum* (detailed in Table 1 and Supplementary Table S2).

**Figure 2:**
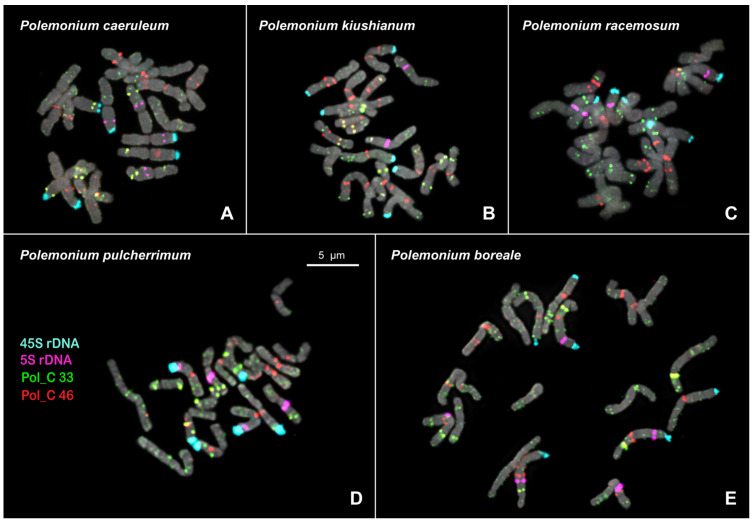
FISH-based localization of 5S rDNA (purple), 45S rDNA (aqua), and also satellite repeats Pol_C 33 (green) and Pol_C 46 (red) in the metaphase spreads of *Polemonium caeruleum* (**A**), *P. kiushianum* (**B**), *P. racemosum* (**C**), *P. pulcherrimum* (**D**), and *P. boreale* (**E**). DAPI-staining – grey. The correspondent probes and their pseudocolors are specified on the left. Bar—5 μm.

The BLAST (version 2.16.0) analysis revealed interspecies homology between most satDNAs identified in genomes of *P. caeruleum, P. pulcherrimum, P. boreale*, and *P. villosissimum* (Figure 2, Table 1).

According to BLAST, five most abundant satDNAs identified in *P. pulcherrimum* (Pol_P 37, Pol_P 67, Pol_P 60, Pol_P 64, and Pol_P 58) and *P. boreale* (Pol_B 26, Pol_B 43, Pol_B 30, Pol_B 63, and Pol_B 53) demonstrated a high degree of sequence identity (89–100%) and similar repeat lengths with five satDNAs (Pol_C 33, Pol_C 46, Pol_C 67, Pol_C 70, and Pol_C 125) from the *P. caeruleum* genome. In *P. villosissimum*, four satDNAs (Pol_V 29, Pol_V 39, Pol_V 66, and Pol_V 131) demonstrated a high degree of sequence coverage/identity with four corresponding *P. caeruleum* repeats (Pol_C 33, Pol_C 46, Pol_C 67, and Pol_C 142) (Table 1, Supplementary Table S2).

SatDNA Pol_B 165 (*P. boreale*) showed sequence similarity (47%/78% coverage/identity) with Pol_C 140 (*P. caeruleum*), and also the sequence of Pol_V 131 (*P. villosissimum*) demonstrated 99%/95% of coverage/identity with Pol_C 142 (*P. caeruleum*). However, in *P. pulcherrimum*, any repeats having sequence identity with Pol_C 140 or Pol_C 142 (*P. caeruleum*) were not detected. At the same time, the genome proportions of two satDNAs of *P. caeruleum* (Pol_C 70 and Pol_C 125) were significantly less if compared with the corresponding homologous repeats identified in genomes of *P. pulcherrimum* and *P. boreale* (Table 1, Supplementary Table S2).

Within the available NCBI database, the sequence homology of the satDNAs identified in genomes of *P. caeruleum*, *P. boreale*, and *P. villosissimum* with tandem DNA repeats from other species was not revealed. At the same time, Pol_P 39 (*P. pulcherrimum*) showed 71–72% of identity/46–47% of coverage with the *Malus sylvestris* genome assembly, chromosomes 2, 5, 6, 10, 13, 15, and 16, and *Rubus chamaemorus* genome assembly, chromosomes 17 and 21.

### 2.2. Chromosomal Localization of Tandem DNAs

For the first time, we carried out a FISH-based chromosomal localization of the marker tandem DNA repeats of *P. caeruleum* (Pol C 33 and Pol_C 46) and also 45S rDNA and 5S rDNA in karyotypes of the species from the *P. caeruleum* complex (*P. caeruleum*, *P. kiushianum* Kitam., and *P. racemosum* (Regel) Kitam.) and *P. pulcherrimum* complex (*P. pulcherrimum* H. and *P. boreale* Adams). A high degree of sequence identity/coverage of Pol_C 33 (508 bp), Pol_P 37 (500 bp), Pol_B 26 (507 bp), and Pol_V 29 (507 bp) and also Pol_C 46 (191 bp), Pol_P 67 (192 bp), Pol_B 43 (193 bp), and Pol_V 39 (192 bp) revealed in genomes of the studied species, allowed us to use satDNAs Pol_C 33 and Pol_C 46 as probes for FISH assays.

**Figure 3:**
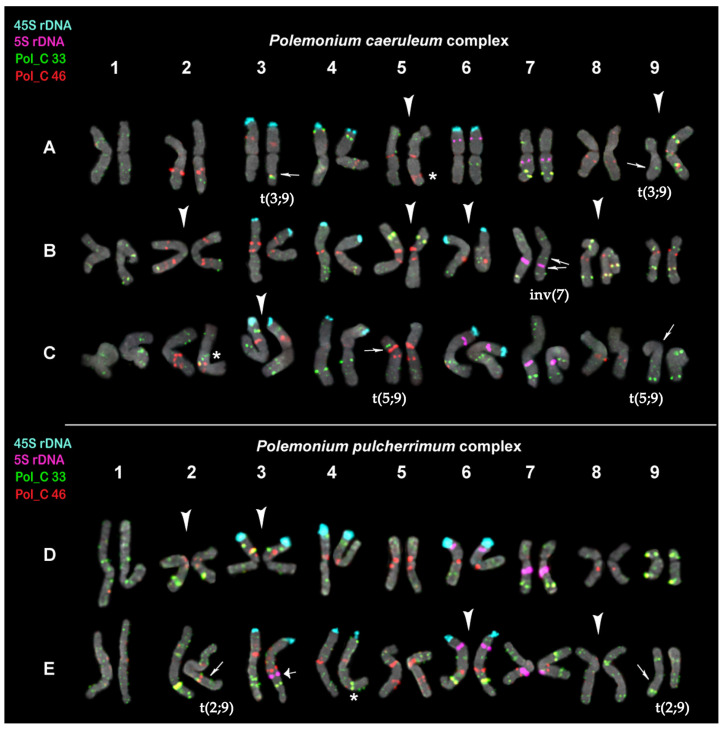
Karyograms of *Polemonium caeruleum* (**A**), *P. kiushianum* (**B**), *P. racemosum* (**C**), *P. pulcherrimum* (**D**), and *P. boreale* (**E**) after FISH with 5S rDNA (purple), 45S rDNA (aqua), and also satellite repeats Pol_C 33 (green) and Pol_C 46 (red) (the same metaphase plates as in Figure 2). The species-specific variants of chromosome localization of Pol_C 33 and Pol_C 46 clusters are shown with arrowheads. Thin arrows point to the assumed reciprocal translocations and paracentric inversion. A thick arrow points to the 5S rDNA locus observed on one homolog of chromosome pair 3 in *P. boreale*. Asterisks indicate polymorphic sites. The correspondent probes and their pseudocolors are specified on the left.

Based on the chromosome morphology and patterns of chromosomal distribution of 45S rDNA, 5S rDNA, Pol_C 33, and Pol_C 46, karyograms of the studied species from the *P. caeruleum* complex and *P. pulcherrimum* complex were constructed (Figure 2 and Figure 3). It was revealed that the karyotypes of all these *Polemonium* species contained nine pairs of metacentric and submetacentric chromosomes which are generally similar in chromosome morphology (Figure 3).

The localization of 45S and 5S rDNA loci in karyotypes of the studied species from the two *Polemonium* complexes was rather similar—45S rDNA clusters were localized on the short arms of chromosome pairs 3, 4, and 6 (Figure 3). In one plant of *P. racemosum* (*P. caeruleum* complex), we observed only five 45S rDNA hybridization signals on chromosomes (on both homologs of 3 and 6 pairs and one homolog of chromosome pair 4) (Supplementary Figure S1).

Clusters of 5S rDNA were observed in the proximal region of the long arms of chromosome pair 7. Moreover, 5S rDNA clusters were detected in the short arms of chromosome pair 6 in all the species except *P. kiushianum* (Figure 3). In the karyotypes of about a fourth of the studied *P. pulcherrimum* plants, the heteromorphism of chromosome 7 homologs in signal size of 5S rDNA loci was observed (Supplementary Figure S1). In the karyotypes of about a third of the studied *P. boreale* plants, 5S rDNA loci were detected in the proximal region of the long arms of one homolog of chromosome 4 (Figure 3E).

Pol_C 33 presented multiple large and small clusters localized in all chromosomes mainly in the interstitial and subtelomeric regions. The satDNA Pol_C 46 was localized in the pericentromeric and/or proximal regions of most chromosomes. Pol_C 33 and Pol_C 46 were often visualized in the DAPI-positive chromosome regions (Supplementary Figure S1). On several chromosomes, colocalization of Pol_C 33 and Pol_C 46 signals was observed (Figure 2 and Figure 3).

The interspecies differences in chromosome distribution patterns of the hybridization signals of both markers Pol_C 33 and Pol_C 46 were found (Figure 2 and Figure 3). In the karyotype of the *P. caeruleum* specimen, heteromorphism of homologs in localization of Pol_C 33 and Pol_C 46 was revealed on chromosomes 3 and 9. One homolog (metacentric) of chromosome 9 presented a rearranged variant of this chromosome revealed previously [23]. The second homolog (submetacentric) differed from the other previously identified normal variant of chromosome 9 [23] by a new reciprocal translocation t(3;9) (Figure 3A). On chromosome 5, a specific variant of probe localization and morphology was revealed. In karyotypes of *P. kiushianum*, we observed the specific variants of probe localization on chromosomes 2, 5, 6, and 8; a paracentric inversion was also detected on one homolog of chromosome pair 7 (Figure 3B). In karyotypes of *P. racemosum*, heteromorphism of homologs in localization of Pol_C 33 was revealed on chromosomes 5 and 9, which could be related to the reciprocal translocation t(5;9). A specific variant of probe localization on chromosome 3 was also observed (Figure 3C). In the karyotypes of *P. pulcherrimum*, specific variants of chromosomes 2 and 3 having large colocalized clusters of Pol_C 33 and Pol_C 46 were detected (Figure 3D). In the karyotype of *P. boreale*, the on-specific variants of probe localization, chromosome 6 with large colocalized clusters of Pol_C 33 and Pol_C 46, as well as on chromosome 8 having mainly Pol_C 33 signals, were found. On chromosome 4, a polymorphyc probe localization was observed. Moreover, heteromorphism of homologs in localization of Pol_C 33 was revealed on chromosomes 2 and 9, which could be related to the reciprocal translocation t(2;9) (Figure 3E).

Thus, in the karyotypes of the studied species, the common patterns of chromosome distribution of 45S rDNA, 5S rDNA, as well as satDNAs Pol C 33 and Pol_C 46 were observed. At the same time, species-specific variants of probe localization on chromosomes as well as chromosome rearrangements were also detected.

## 3. Discussion

Most *Polemonium* species are distributed exclusively in Eurasia and North America, and some species have overlapping ranges. Tall plants species of *P. caeruleum* (Eurasia and North America), *P. kiushianum* (=*P. caeruleum* ssp. *kiushianum* (Kitam.) H. Hara) (N. China to Korea, Japan), and *P. racemosum* (=*P. liniflorum* V.N. Vassil) (E. Siberia, Russian Far East to China, Mongolia, and N. Japan) are included in the *P. caeruleum* complex [8]. The *P. pulcherrimum* complex contains the dwarf species *P. pulcherrimum* (E. Siberia to Russian Far East, W. USA), *P. boreale* (growing mainly in the subarctic and subalpine zones in Eurasia and the USA), and *P. villosissimum* (Alaska, USA) [8]. *P. villosissimum* was previously considered as a subspecies of *P. boreale*. However, it has recently been recognized as a separate species [24]. The identification of *Polemonium* taxa, including those within the *P. caeruleum* complex and *P. pulcherrimum* complex, is ambiguous due to the complexity of their morphological evolution [4]. Intermediate forms are often found within and between the complexes [1,2,3], and systematic and phylogenetic studies of the species of this genus as a whole [8,9,15] would be advanced through additional research of their repeatomes as well as chromosomal organization of their genomes.

In plants, repetitive DNA sequences can make up to 95% of the genome, and they play an important structural and functional role and also contribute to the process of speciation [25,26]. In the present study, a comparative bioinformatic analysis of repeatomes of *P. caeruleum* (*P. caeruleum* complex) and also *P. pulcherrimum*, *P. boreale*, and *P. villosissimum* (*P. pulcherrimum* complex) revealed significant similarity in the distribution of various types of DNA repeats, which is consistent with the close relationship of these species reported earlier [8,9].

It is known that the most common transposable elements (TEs) of plant repeatomes are retrotransposons (class I) [26,27,28]. These TEs make a significant contribution to the genome DNA content and diversity during the speciation [28,29,30]. In the studied *Polemonium* species, mobile elements of class I also constituted a large proportion of their repetitive DNA (59.20–66.08%). Moreover, Ty3-Gypsy retroelements were almost 1.7–2.4 times more abundant compared to Ty1-Copia elements. At the same time, in the genome of *Gilia yorkii* Shevock & A.G. Day (a species from another genus of the *Polemoniaceae* family, the genome assembly of which was previously presented), class I transposable elements constituted 45.81% of the entire genome assembly, and the content of Ty1-Copia elements was 1.8 times higher than that of Ty3-Gypsy elements [31]. The evolutionary dynamics of individual LTR retrotransposons might differ between retrotransposon families and plant species, probably due to the interactions of various genomic and environmental factors [32,33,34,35]. For example, within the legume family, the composition of repeats of species from different genera can vary due to the predominance of Ty1-Copia elements [36,37] or Ty3-Gypsy [38,39].

The process of speciation may also be accompanied by dynamic changes in the repetitive fraction of DNA, including satellite DNA [40,41,42]. In different satellite DNA families, a high rate of genomic changes was revealed, and satDNAs can be either species-specific or common to a certain group of related species [43,44]. In the present study, despite the fact that the number of identified satDNAs varied among *P. caeruleum*, *P. pulcherrimum*, *P. boreale*, and *P. villosissimum*, the main set of common tandem DNA repeats was similar (sequence identity 87–100%), and their monomeric sequences were mostly identical in length. The phylogenetic analysis of the genus *Polemonium* based on AFLP data, revealed that the species from the *P. pulcherrimum* clade are more closely related to the members of the *P. caeruleum* than to the species from other complexes [8]. In the repeatomes of the studied species from both *P. caeruleum* complex and *P. pulcherrimum* complex, quite a large number of common satellite repeats were found. Our data demonstrate a close relationship between the genomes of the species from these two *Polemonium* complexes. Moreover, the revealed high degree of homology of satellite sequences, which are considered to be evolutionarily rapidly changing fractions of the genome, indicates recent divergence of these species from the common ancestor genome. These results support current understanding of the taxonomy of *Polemonium*, suggested rapid genus radiation ca. 7.3–10.8 Ma [45,46].

SatDNA was shown to be involved in key processes of the formation of critical chromosomal structures, such as DNA packaging and chromatin condensation. SatDNA is often associated with heterochromatin and chromosome rearrangements [19,47,48]. Currently, satDNA distribution patterns on chromosomes are widely used as chromosomal markers for identifying chromosomes and subgenomes in karyotypes of diploid and polyploid plants, detection of chromosomal rearrangements, and also for studying the pathways of chromosomal evolution of related taxa [20,49,50,51,52]. In this study, two most abundant common satDNAs, Pol_C 33 and Pol_C 46, provided informative localization patterns in all chromosomes, and these satDNAs were used as markers for chromosome pair identification to perform a comparative karyotype analysis.

In plants, the process of speciation is associated with variability in the number of gene copies in 45S and 5S rDNA clusters, as well as the number of such clusters in the genome and their localization on chromosomes [53,54,55,56]. These characters can vary not only between the species but also within them [53,54,55,56]. However, the high conservative nature of ribosomal genes as well as their localization in the genome allows them to be classified as synapomorphic traits, making it possible to determine common origin and genomic relationship [56,57]. In this study, the comparative analysis of repeatomes demonstrated that the 45S rDNA and 5S rDNA content in the genomes of the studied *Polemonium* species varied only slightly. FISH analysis also revealed that the karyotypes of four species of the five studied taxa contained three pairs of chromosomes carrying 45S rDNA clusters and two pairs of chromosomes bearing 5S rDNA loci. The exception was *P. kiushianum*, whose karyotype contained only one pair of chromosomes with 5S rDNA. The similarity in chromosome numbers (2*n* = 2*x* = 18) and morphology as well as in chromosome distribution patterns of 45S rDNA and 5S rDNA clusters could be related to the common origin of the studied species from both *Polemonium* complexes.

The revealed similarity in the distribution patterns of the main sites of 45S rDNA, 5S rDNA, and also satDNAs Pol_C 33 and Pol_C 46 on chromosomes of the studied *Polemonium* species allowed us to construct their karyograms in accordance with the karyogram of *P. caeruleum* var. Belosnezhka reported earlier [23]. All these molecular cytogenetic markers allowed us to identify the chromosome pairs, analyze the structure of karyotypes, as well as reveal various chromosome rearrangements in the studied species of both *Polemonium* complexes. The obtained results allowed us to establish the cause of the morphological diversity of chromosome 9 in the karyotypes of different *Polemonium* species, previously reported [17,23]. Moreover, the observed interspecies diversity in the intensity and position of studied molecular cytogenetic markers observed on the chromosomes of different *Polemonium* species demonstrated both species-specific karyotypic features and chromosomal rearrangements that occurred during the process of divergence from a common ancestor. It is known that chromosomal rearrangements often accompany plant evolution [20,49,58,59]. The results of molecular phylogenetic analysis of *Polemonium* based on nrDNA and ptDNA, and also AFLP data, resulted in conflicting versions on phylogenetic reconstruction of the genus *Polemonium* [8,9]. Finally, it was suggested that the monophyletic origin with further complex evolutionary history of the genus *Polemonium*, which included rapid radiation, repeated acts of interspecific hybridization, as well as translocations and chromosome inversions during speciation [2,8,9]. Our results could be cytogenetic evidence for these phylogenetic suggestions.

The species *P. pulcherrimum* and *P. boreale* from the *P. pulcherrimum* complex are clearly distinguished by their morphological features [1,2,3]. In our study, the interspecies differences in chromosome distribution patterns of Pol_C 33 and Pol_C 46 demonstrated that karyotypes of *P. pulcherrimum* and *P. boreale* underwent reorganizations during speciation. The *Pulcherrimum* clade was divided into two subclades. One of the subclades includes *P. pulcherrimum*, and the other one comprises *P. boreale* [8]. At the same time, the previous AFLP results demonstrated the monophyletic origin of the species from the *P. pulcherrimum* complex. The *P. pulcherium* clade is more closely related to members of *P. caeruleum* clade than to the species from other *Polemonium* complexes [8].

The identification of these taxa is difficult due to the high level of intraspecific variability of morphological characteristics [3,14,60]. The species *P. caeruleum*, *P. kiushianum*, and *P. racemosum* (the *P. caeruleum* complex) are quite similar in morphological features. Moreover, between closely related species within the genus *Polemonium*, the hybridization events were detected [3,12,14]. In Eastern Siberia, intermediate forms between *P. racemosum* and *P. caeruleum* (*P. caeruleum* complex) were found in the overlapping areas [3,60]. In Japan, the species from the same complex, *P. caeruleum* and *P. kiushianum*, can also occupy similar habitats. At the same time, RAPD (random amplified polymorphic DNA) analysis showed that these species do not hybridize in their overlapping habitats [13]. *P. kiushianum* is known to be a rare, endangered relict species that occupies a very narrow habitat, which is considered either a subspecies of *P. caeruleum* or an independent species [13,14,61,62]. In the present study, different chromosome patterns of the cytogenetic markers observed in karyotypes of the studied species (e.g., variants of chromosome 4) indicate that *P. kiushianum* is an independent species rather than a subspecies of *P. caeruleum*.

Thus, our comprehensive comparative study of the repeatome composition and also FISH chromosome distribution patterns of 45S rDNA, 5S rDNA and satDNAs, Pol_C 33, and Pol_C 46, in karyotypes of the species from *P. caeruleum* complex and *P. pulcherrimum* complex of the *Polemonium* genus revealed a similarity between their genomes, which indicates a common origin of these species. Moreover, the interspecies diversity in the intensity and position of studied molecular cytogenetic markers observed on the chromosomes of different Polemonium species demonstrated both species-specific karyotypic features and chromosomal rearrangements that occurred during the process of divergence from a common ancestor. Our findings could be cytogenetic evidence for phylogenetic data on the monophyletic origin and further complex evolutionary history of the genus *Polemonium*, which included hybridization events and genome reorganizations during speciation [8,9]. Furthermore, the peculiarities of chromosome patterns of 45S rDNA, 5S rDNA, Pol_C 33, and Pol_C 46 in the karyotype of *P. kiushianum* confirmed its taxonomic status as an independent species. Our findings show that the developed approach may be useful for further studies of chromosome organization in karyotypes of other species within the genus *Polemonium*.

## 4. Materials and Methods

### 4.1. Plant Material

Seeds of five *Polemonium* species (detailed in Table 2) were obtained from the germplasm collection of All-Russian Institute of Medicinal and Aromatic Plants (AIMAP), Moscow, Russia.

### 4.2. Sequence Analysis and Identification DNA Repeats

For genome-wide comparative analyses of *P. caeruleum* [23] and the species of the *P. pulcherrimum* complex sp., the publicly available sequencing (Illumina platform) data of *P. pulcherrimum* (sample ERR5529402; https://www.ncbi.nlm.nih.gov/sra/ERR5529402 (accessed on 30 April 2024)), *P. boreale* (sample ERR5529630; https://www.ncbi.nlm.nih.gov/sra/ERR5529630 (accessed on 30 April 2024)), and *P. villosissimum* (sample ERR5529443; https://www.ncbi.nlm.nih.gov/sra/ERR5529443 (accessed on 30 April 2024)) were used. Information on library preparation (i.e., whether PCR amplification was used) is not available for the NCBI/SRA datasets analyzed. Therefore, abundance estimates of repetitive elements should be interpreted with caution.

From basecalled sequencing data, 9,005,690 of paired-end reads (100 bp in length) (*P. pulcherrimum*), 10,712,572 of paired-end reads (100 bp in length) (*P. boreale*), and 10,789,070 of paired-end reads (100 bp in length) (*P. villosissimum*) were selected and filtered by quality. Then, 1,682,289 (*P. pulcherrimum*), 1,708,789 (*P. boreale*), and 1,638,620 (*P. villosissimum*) high-quality reads were randomly selected for further analyses using RepeatExplorer2/ TAREAN pipelines based on the Galaxy platform (https://repeatexplorer-elixir.cerit-sc.cz/ galaxy/, 26 April 2025) [63,64], and it was within the limits recommended by the developers of these programs (genome coverage of 0.01–0.50× was recommended) [61]. Due to the limited information on the genome sizes of the *Polemonium* species within the Plant DNA C-values Database [65,66], we used the genome size data of the only species available in the databases, *P. reptans* L. (6.05 pg), which contained the same chromosome number (2*n* = 18) [17,66].

The sequence homology of the identified tandem DNA repeats from *P. caeruleum, P. pulcherrimum, P. boreale,* and *P. villosissimum* (Supplementary Table S2) was estimated using the Basic Local Alignment Search Tool (BLAST, version 2.16.0) (NCBI, MD, USA) (Table 1).

Based on two abundant tandem DNA repeats of *P. caeruleum* (Pol_C 33 and Pol_C 46), oligonucleotide FISH probes were generated (Supplementary Table S3) using Primer3-Plus software (https://www.primer3plus.com, accessed on 12 April 2025) [67].

### 4.3. Chromosome Spread Preparation

Seeds of the studied *Polemonium* species were germinated in the Petri dishes for 3–5 days at room temperature (RT). Root tips (0.5–1 cm long) were kept in ice water for 24 h for the accumulation of mitotic cells and then fixed in ethanol/acetic acid fixative (3:1) for 2–3 days (RT). The fixed roots were put into 1% acetocarmine solution (in 45% acetic acid) for 20–30 min. On the glass slide, the meristem was cut off from the root tip, macerated in a drop of 45% acetic acid, and a squashed preparation was made using a cover slip. The slide was frozen in liquid nitrogen, dehydrated in 96% ethanol, and air dried.

### 4.4. FISH Procedure

Four DNA probes were used in FISH assays. Two wheat probes, pTa71 (including 18S-5.8S-26S (45S rDNA)) and pTa794 (including 5S rDNA) [68,69], were labeled directly with fluorochromes Aqua 431 dUTP and/or Red 580 dUTP (ENZO Life Sciences, Farmingdale, NY, USA) by nick translation according to the manufacturer’s protocols. Two oligonucleotide probes, Pol_C 33 and Pol_C 46, were synthesized and labeled with Cy3-dUTP and/or 6-FAM-dUTP in Syntol (Moscow, Russia) (Supplementary Table S3).

The FISH procedure was performed according to the protocol reported previously [70]. The chromosome slides were pretreated with RNase (Roche Diagnostics, Mannheim, Germany) (1 mg/mL in 2 × SSC) at 37 °C for 1 h. Then, the slides were washed three times in 2 × SSC for 10 min each, dehydrated in the graded ethanol series, and air dried. The labeled probes (40 ng each) were dissolved in a hybridization mixture (containing 50% formamide) in a total volume 15 μL, dropped on the slide, sealed with rubber cement under coverslips, and co-denatured at 74 °C for 4 min. After overnight hybridization at 37 °C, the slides were washed in 0.1 × SSC and then in 2 × SSC (5 min at 42 °C each), followed by a 5 min wash in PBS (RT), dehydrated in the graded ethanol series, and air dried. Then, the slides were stained with 0.1 μg/mL DAPI (4′,6-diamidino-2-phenylindole) (Serva, Heidelberg, Germany) dissolved in Vectashield mounting medium (Vector laboratories, Peterborough, UK) under coverslips.

### 4.5. Analysis of Chromosome Preparations

The chromosome slides were analyzed using the Olympus BX 61 epifluorescence microscope equipped with a standard narrow band-pass filter set (Olympus, Tokyo, Japan). From each sample, at least five plants and fifteen metaphase plates from each plant were analyzed. Images were acquired with a monochrome charge-coupled camera (Cool Snap, Roper Scientific, Inc., Sarasota, FL, USA) and processed using Adobe Photoshop 10.0 software (Adobe, Birmingham, AL, USA).

Chromosome pairs in karyotypes were identified according to the chromosome morphology and localization of the studied chromosome markers. In the karyograms, chromosome pairs were set in the decreasing order of size.

## Figures and Tables

**Table 1:** The satDNAs identified in genomes of *P. caeruleum*, *P. pulcherrimum*, *P. boreale*, and *P. villosissimum* demonstrating the sequence similarity (coverage/identity) according to BLAST.

SatDNA/Genome Proportion, %/Repeat Length, bp/Homology with SatDNAs *P. caeruleum*			
*P. caeruleum* ^1^	*P. pulcherrimum*	*P. boreale*	*P. villosissimum*
Pol_C 33/0.44/508	Pol_P 37/0.3/500 92%/91% of coverage/identity with Pol_C 33	Pol_B 26/0.36/507 100%/89% of coverage/identity with Pol_C 33	Pol_V 29/0.31/507 99%/87% of coverage/identity with Pol_C 33
Pol_C 46/0.24/191	Pol_P 67/0.1/192 99%/98% of coverage/identity with Pol_C 46	Pol_B 43/0.19/193 95%/99% of coverage/identity with Pol_C 46	Pol_V 39/0.18/192 99%/99% of coverage/identity with Pol_C 46
Pol_C 67/0.12/89	Pol_P 60/0.11/89 85%/94% of coverage/identity with Pol_C 67	Pol_B 30/0.3/89 90%/100% of coverage/identity with Pol_C 67	Pol_V 66/0.046/89 100%/100% of coverage/identity with Pol_C 67
Pol_C 70/0.092/393	Pol_P 64/0.1/393 97%/97% of coverage/identity with Pol_C 70	Pol_B 63/0.1/393 100%/100% of coverage/identity with Pol_C 70	-
Pol_C 125/0.016/83	Pol_P 58/0.13/83 89%/100% of coverage/identity with Pol_C 125	Pol_B 53/0.13/83 100%/100% of coverage/identity with Pol_C 125	-
Pol_C 140/0.012/187	-	Pol_B 165/0.012/97 47%/78% of coverage/identity with Pol_C 140	-
Pol_C 142/0.012/168	-	-	Pol_V 131/0.015/168 99%/95% of coverage/identity with Pol_C 142

^1^ These data were taken from our previous study [23].

**Table 2:** List of the studied *Polemonium* accessions.

Polemonium Species	Voucher/Origin
*P. boreale* Adams	K 22729-23/Russia, Republic of Sakha (Yakutia) Yakutsk, Botanical Garden of the Ammosov North-Eastern Federal University, 2021
*P. caeruleum* L.	K 3345-02/Russia, Moscow region, 2023
*P. kiushianum* Kitam.	K 246-20/Germany, Bonn, Botanical Garden, University of Bonn, 2020
*P. pulcherrimum* H.	K 72-22/Russia, Republic of Mari El, Yoshkar-Ola, Botanical Garden of the Volga State University of Technology, 2019
*P. racemosum* (Regel) Kitam.	K 22729-23/Russia, Republic of Sakha (Yakutia) Yakutsk, Botanical Garden of the Ammosov North-Eastern Federal University, 2022

## Data Availability

The original contributions presented in this study are included in the article/Supplementary Materials. Further inquiries can be directed to the corresponding author.

## Supplementary Materials

The following supporting information can be downloaded at https://www.mdpi.com/article/10.3390/ijms27010229/s1.
