# Supplementary material for: Comparison of Genomes of Species from Polemonium caeruleum Complex and Polemonium pulcherrimum Complex Based on Repeatome and Chromosome Analysis
Source: Int J Mol Sci. 2025 Dec 25;27(1):229. doi: 10.3390/ijms27010229 (PMC12785893; doi:10.3390/ijms27010229)
Supplement: Supplementary file 1 [file ijms-27-00229-s001.zip › ijms-4025810-supplementary.pdf]

Supplementary Material

Table S1. Proportions of major DNA repeats identified in genomes of *P. caeruleum, P. boreale, P. villosissimum* and *P. pulcherrimum*.

Repeat Name	Genome Proportion, %			
	***P. caeruleum*[1]**	***P. pulcherrimum***	***P. boreale***	***P. villosissimum***
Retrotransposons (Class I)	66.08	55.33	61.03	59.20
Ty1 Copia	19.25	19.91	16.85	16.93
Ale	0.24	0.25	0.59	0.25
Angela	7.66	8.21	6.41	7.22
Ikeros	0.06	0.06	0.1	0.1
SIRE	10.70	10.8	8.99	8.78
TAR	0.31	0.31	0.44	0.25
Tork	0.28	0.28	0.32	0.33
Ty3 Gypsy	44.46	33.96	41.22	39.20
non-chromovirus Athila	10.90	12.20	11.33	8.47
non-chromovirus Tat	0.86	0.20	0.91	0.83
chromovirus CRM	0.40	0.31	0.40	0.28
chromovirus Galadriel	0.06	-	0.04	0.06
chromovirus Tekay	32.19	21.25	28.53	29.54
chromovirus Reina	0.05	-	0.01	0.02
LINE	0.08	0.09	0.26	0.17
Unclassified ME Class I	2.29	1.37	2.70	2.90
DNA Transposons (Class II)	0.57	0.09	0.6	0.13
CACTA	0.17	-	0.06	0.06
hAT	0.04	-	0.1	-
MuDR_Mutator	0.36	0.09	0.44	0.07
rDNA	0.42	0.74	0.64	0.54
Unclassified repeats	3.31	6.67	6.86	5.26
Satellite DNA	0.87	1.64	0.68	0.17
Low copy DNA sequences	28.75	35.43	30.19	34.7
Putative satDNA families	6 high confident<br>3 low confident	6 high confident<br>5 low confident	5 high confident<br>7 low confident	3 high confident<br>8 low confident

[1] These data were taken from our previous study [23].

Table S2. Characterization of the tandem DNAs revealed in repeatome of *P. pulcherrimum, P. boreale* and *P. villosissimum* using RepeatExplorer2/TAREAN.

***P. pulcherrimum* putative satellites (high confidence)**

Tandem Repeat/ Genome proportion [%]	Repeat length, bp	Sequence
Pol _P 16/0.59	178	CTTTTAACTTCTAGGCTTCGTACTGATATGACAGAACATCGTATCTCAACA<br>CAGTCAACAAGGAGAGGATGCGTGTCGAAAAGAGCTTTCCATATTGGGAT<br>AATAGTAACAATTCTGAGACCCGTAGCAAAGGTTATGGCCAAAAGACTAA<br>AACACCCTCAGATCCGTGAACATAACA
Pol _P 37/0.3	500	GTAGAAATTGAGTGCCTTGATGTGGTCACTAACATGCACACTACAAAAGA<br>AAATAAAAATTACCAAATTATATGGAAACACATTTACCCTAAGTGGATGA<br>AACCCATAACAATCATCTAGAGTGTTGACATAAGAGAATCCCTTGCACATG<br>AGTGAATCCCTTAGATCATTCAATGCATATGGAATCCAAATGAGAGTCCAT<br>GTCTTAGGCCAAAGACATCATGTTGTTGTTGATGCTTGAGGTTTAGTGCAT<br>CATACTGGTGAATGAGATACATCGATAGGTGGTGGTGGTGAACACCCAAT

		TGAGTTCAATCTTCACTATTGTACAAGTAAATGTCACAAGGTATGTTTCAA<br>AACCACTTATGTCATGTAGCAAAGAATTAAATGCACAATGCCATAAGTTGC<br>TTCAATTCCTATGTGAAGTTGTGGAGGTAAGTGTAAACATTATGGCTGTAA<br>ACTATAACATTTTGGATGAAAATAGTTACTTTTATGGTTTTTTT
Pol _P 58/0.13	83	GTAGTGGTAGTTGTATTTGGACGTTAATAGTCATGGAGTTCTCTCTTAGCTT<br>GTAGTGAGATATGTGAACTTACTGAAATGTA
Pol _P 60/0.11	89	TTGGGCTCAAAAGAGGCCCATTAAAAATTAATGACCCATTAGTAAGATATT<br>TATCATTTTTATTTCAAAAAAACATGTTTATTTTTTGT
Pol _P 64/0.1	393	TCAAATGAACATCACAAATATACCTACCTTCGCATATTGTTAGGCTAAGAA<br>TCACCTAATACATGTTTTAATATCCGTTACTTAGTATGTACAATGTTGGAAC<br>ATAAAAGTCCATAGTGAGGTATGAAATAAATGAACATAAATATAAGCCAT<br>TTGTACGTTTCGTAGTCCCACACTAACCTTTCTTATGTTTCTATAACCATTA<br>CGTAGAATGTCCATGTGTATAAAACTAGAATCTATCTCACAAGAAGGACC<br>ATAATGATAGTTATGTGACAACACTAAACTAAATTAGTAATAATTACAAGT<br>CACATAGTTGGTGGTGTTAGGATCTATCGATTCTTAACCCCACATGCATGA<br>AGTTGAGAACTTGTACGACGATAAGTACCGGGATTG
Pol _P 77/0.058	69	TAAAAACACTAGAAAACATTAGAAACCCTAAGGTCACTAAAAACATTAAT<br>GTTTTTCCCCCGAGGACAC
		P. pulcherrimum *putative satellites (low confidence)*
Pol _P 39/0.28	3831	AGACTTATTTTCCAGAAGTACTAGATGAAAACAAAATCTAAAATACAGCTT<br>TAAGATTGTTAGAAATCTGGTATATAAATCTGAAAATATAGAAGCAACAA<br>AGACGTAAATAACTTTAACGCACATAAATTAAAACTCACGGATTGAAAGT<br>CGTCTGAAGCGTATCGTTAAATGGAAGACAGTCTCGTTAATACGATCTCCT<br>TAAAGCTGTATTGCCCCCCACGCATCGGTGCTTTATTTATGGTTCAAGGGC<br>AACCACTTCCCAGGATAAAAGCTTCCCCGTATAACGACGAAAGCACTTAGT<br>CGTTATACGCAACGAACTAACTATGCTAAAGAGATTTAGATAGTAGAATG<br>ATTGTTCCATAGGATGATTTGATGAAATGAGAGGAGACAACTATTTATAGA<br>GATGGAGGTGGCTATTCAGAATAGACACAACTCTTGGTTTGATTCTAACAG<br>TTGCAACTGTTCATTCGAACTGAATAGTTGTGACTATTCAATTCAAATTCA<br>AACCAACTAGTTTCTAGCTCAAACATAATTCCAACCAACCTACGTTCACGA<br>GCCGACCGGTCGAATAGCATTGCCGCACACGTGTTGTTTGGAATTTGTCAA<br>ACACTCCCTTTATCTATAAAATTGCTACGCCTAATGGGACGCACAATTTTC<br>GAATGAGAAACACCTCATTATAACGTCTCATTTTTCATTCATCACTAAGTT<br>GATTTTAATGGTTGACGTTTCAAATCGTTCTCTTATATTCATAATTTACGAT<br>GAACGTTGTGAACTCTTACTTGAGCTCTCTCTTGAAACCGGTTCAAAACCA<br>TATTGGAAATCAACTTTTCCAACAATCCCCCACATGAATGACAAATTAGCG<br>TTTTACTGCTGAAAATGTCTGTTTCGGAATGAAATCTCTTAAGCGGTGACT<br>AATTGCTTAAAGAAAGGTAGCCATAGACTTTGAACCTTCCTGTTAGTGATC<br>TCTATAGGGCATACTGGATTGTCAGTGAGCGATAGTGCGCCTTGAACTGCT<br>TTCCGTGGTGTATGCCTAGTCGATAGTAACCACACAATTAATTACCTGAAC<br>ATAGTCAGTTCTTATGGTTGTGTTCATTTTGGCCTTGAAACACTGTCCTGGT<br>TTCATGAGTAGACTTAGAGGAGTGTAGCCTCGTCATCCTCCTAGAACCGGC<br>CCGTTCCTACTCATATAGGTGAGTTCATTAAGAGTATTCTCCATACCCCTCT<br>TAAATTTCCCTTTAAGATATGACCTCATTAAAAGCACTTGGCTTATCCTGTT<br>CCGATCACTCATAGTCGTCCTAGGAATGGGATGAAAACGTTGTTTTCCGGT<br>GAGTGATATTTGATTTGCATCTGGTAGTTGGGTTGTCCCTTTTGAACCTAGT<br>TCTTGGGATCTCCAGTCACCTAGGTTGGGTTTTCCGCTACGTGCAACTCTTA<br>CGAGATAGGCTTTAGCCCCATTCCTCTTGACGAACTTGCAACTAGTTCTCTC<br>GATAAACCTTTAGTCAACGGATCCGCTATATTTTCTTTTGACTTTACATAAT<br>CGATCGATATTATACCACGTGAGAGAAGTTGCCTAATGGTATTATGTCTAC<br>GACGTATGTGTCGAGACTTACCATTATATATGCTACTTTGTGCCCTTCCTAT<br>TGCTGATTGACTATCGCAGTGAATGCAGATAGGAGGTACTGGTTTTGGCCA<br>TGGTGGAATATCTTCGAGGAAGTGACGAAGCCATTCTGCTTCTTCCCCTGC<br>CTTGTCTAACGCTATGAATTCTGATTCCATAGTAGACCTTGCAATGCACGT<br>CTGCTTTGAAGATTTCCAGCATACCGCTGCACCACCTAGTGTAAATACATA

		TCCACTGGTGGATTTGGAATCTTGGGTGTCAGAAATCCAGTTTGCATCACA<br>ATACCCTTCCAGAACGGCTGGATAGTTTGAATATTGCAAACTTAGAGTTTT<br>GGTGTATCGTAGATACCTTAGAACTCTCATCAAAGCTGTCCAGTGATCCTG<br>ACCTGGATTGCTTGTAAAGCGACTAAGCTTTCCGACAGCTTGTGTAATGTC<br>CGGCCTTGTACAATTCATGATGTACATCAGGCTTCCGATTATTTGAGCATA<br>TTCTGACTGCTTGACTGCACTACCTCGATTCTTGGTGAGGTGTACACTTACG<br>TCAACTGGTGTCGTAACAGGTTTGCTATTTCCTTTATCAAACTTCTCGAGAA<br>CTTTCTCGATGTAGTGAGATTGGGATAGCGCATAACCATTTGACGTAGCTG<br>ATATTTTGATTCCTAGAATAACGTCAGCTATTCCAAGATCTTTCATATCAAA<br>CTTCTTTGACAATAATTTCTTTGTCGCAAGAATTATTGCCTTGTTGCTTCCA<br>AGAATTAGCATATCGTCGACATATAAGCATATAATGACACATCCTTCGGCG<br>ACGGTTTTGGAGTAAACACACTTGTCGCACTCATTTGTTCGAAACCCATTA<br>GACATCATAATGTTATCGAACTTCTCATGCCATTGTTTCGGGGCTTGTTTTA<br>ATCCATACAAGGATTTCTTCAATCGACAATATTTATTCTCGTTTCCAGCAAC<br>TTTAAAACCTTCAGGTTATTCCATATAGATTTCTTCCTTCAATTCTCCGTTT<br>AGAAACGCAGTTTTGACATCCATTTGATGAATCGCCGCTATTGCTGTAAGC<br>ATGCGGATGGATGTAATTCTCGTAACTGGAGAATAGGTATCAAGGATATTA<br>ATTTCTTCCTTTTATTATAATTACGCATGAATTATTCATTCATGGAATTAAC<br>GTCGAAATAATATTCAAAACTTTGAAGTGTTGTGTGCGAAATGCAAGTATA<br>TACATACTCCGTTTGGTTAAAATAAAATCATAAGTGCTTTAAACTCTTGATT<br>TATTTTACCAACGTCGATGAGTGCTTTGAAACTCTTCGAACTATATACTAG<br>CTACAAAATACATCGTTATTTAGTAATCGACAAATCATAAGTACTTTGATA<br>ACTCTTGATTTGATTGATACTAAATTCTTTCATCCATGAGTGCTTGAAACTC<br>TTGGATTCCACGAGTATTTGGTCAACCATAGCCATGAGTACTTCAAAACTC<br>TTGGAATGTGGACCCCCACAATGCACACGATTTATTTCGGTCATCTCAACC<br>GAAACTTTGAATACGTCAATATTCATGCTTAATGCCTTTTTAAACTAAGGA<br>CTATAGCTTCCATCCGAGCTCTAGTTAGTTTAAAATATTTCATAGGAACAT<br>GAATACTATTATTTCGACCAAGCGCTCAACAATAAATAGATAGCTCTATTT<br>TTGTAGACACATTTAAGAAATTAATTATCAACAACTTTCATGTTTTGACAA<br>CAACGATCCGAGATCGTTATTCTTGTCTAAAATCACAAACTCGGAGATGTG<br>ACCATTACAAATTTCCGTTTCAACACAGAAACTTTGACGCACAATATCTTG<br>AGTTGCATAACTTCAATTATTGCGCCGATTTTTCTCACATATTCATTTTTCA<br>GATTCCTACAACTTTTGTGTTTGGACAAAACTGATATGAAATCGTTATACTT<br>GTCTAAAACAACAAAACTTGAAATCTAGAAAAACAAAATTCTGTTCCAAA<br>ACAGGAACTTTTATGCACAATATCTGGAGTTCTACAACTCCAATTTACATG<br>CCGTTTCTTCTGAAATGTTCTTCTCATTGAGATCTACAACTTTTGTGATTTG<br>ACAAAACCGATATGAAATCGTTATCCTTGTCGAAAACAACAAAAACAGAA<br>TTCTGGTCGTTCCCTTTCTTCGCCAACAGTCGCAATGTTTGGCTAAGGGTAC<br>CCCGACCGGTACAATACACTGAATGTGTATTTCAGTTTC
Pol _P 53/0.17	177	CTTTGTAATTCAATATCTCTTAAACCGTAAGTCGAAAAATCATGAAACTTT<br>ACAGTAGCAATGATACATAGGTGTACTAAATACCCATAAAACAGTTTTGA<br>AAAATGATGAAGGGTTCACGAGTTATAGGCCAAAACATACAGCTGCCCAG<br>TAGGAAATTCTGATCTGTGGTTTCAA
Pol _P 67/0.1	192	ACTTCTAATAATCCATATTGGTCCCACTTCTATTCAAAAGTCAAACTATCTA<br>TATAACAATCTACTATGGGGATTAGTGTATCCCCAAAATATGACAAGTGGA<br>ATAGTTTATCCCGCCTCTTTAATTTACAGTGTAAACCCTTGAATGTGTGGAT<br>GAGAAATTTAGAAATCCAAACAATCCTCTTTTTTTTT
Pol _P 70/0.07	18	CAATTCATAAGTTGAAAG
Pol _P 146/0.016	192	TCTCCATTGCCAAGCCCTCCAATTCTTTCTTCAAAAATAACCACCACCGAC<br>ATATTTACTCTAGCCCTACTGGGAGTAGGGGTGACCAATATCATCTCCATT<br>ACCAAACCCCAAATTTTTACTACAAATATAGCCACCACCAACATATACACT<br>ATCCCAAGTTAATCTTACTGGAGATGATGACCTATATCA
		P. boreale putative satellites (high confidence)

Pol _B 26/0.36	507	ATAAAAGTAAATGTTTTCTTCCAAAATGTTATAGTTTACCGCCATAATGTTT<br>ACACTTACCTCCACAACTTCACATAGAAATTGAAGCAACTTATGGCATTGT<br>GCATTTAATTCTTTGCTACATGACATAAGTGGTTTTGAAACATACCTTGTGA<br>CATTTACTTGTACAATAGTGAAGATTGAATTCAATTGGGTTCACCACCACC<br>ACCTATCGATGTATCTCATTCACCAGTATGATACACTAAACCTCAAGCATC<br>AACAACAACATGATGTCTTTGGCCTAAGACATGGACTCTCATTTGTATTCC<br>ATATGTATTGAATGATCTAAGGGATTCACTCATTTGTTTACATGTGCAAGG<br>GATTCTCTTATGTCAACACTCTAGATGATTGTTATGGTTTTCAGCCACTCAG<br>GGTGAATGTGTTTCCATCTAATTTGGTGATTTTCATTTTCTTTTGTAGTGTG<br>CATGTTAGTGACAACATCAAGGCACTCAATTAGAAAAAAAAAAC
Pol _B 30/0.3	89	GAAATTAATGACCCATTAGTAAGATATTTATCATTTTTATTTCAAAAATAC<br>ATGTTTATTTTTTGTTTGGGCTCAAAAGAGGCCCATT
Pol _B 53/0.13	83	GTAGTGAGATATGTGAACTTACTGAAATGTAGTAGTGGTAGTTGTATTTGG<br>ACGTTAATAGTCATGGAGTTCTCTCTTAGCTT
Pol _B 63/0.1	393	CACATTGACATTCTGCGTAATGGTTATTGAAACATAAGAAAGGTTAGTGTG<br>GGACTACGAAACGTACAAAAGGCTTATATTTATGTTAATTTATTTTATACC<br>TCACTATGGATTTTTATGTTCCAACATTGTACATACTAAGTAAGGGATATT<br>AAAACATTTAATAGGTTATTCTTAGCCTAACAATATGCGAAGGTAGGTATA<br>TTTGTGATGTTCATTTGACAATCCCGGTACATATCGTCGTATAAGTTTCAA<br>CTTCATGCATGTGGGGTTAAGAATCGATAGACCCTAACACCACCAAATATG<br>TGACTTGTAATTATTACTAATTTAGTTGAGTGTTGTCACATAACTTTCATTA<br>TAGTCCTTCTTGTGAGATAGATTCTAGTTTTATA
Pol _B 183/0.0099	69	CATTAATGTTTTTAGTGACCTTAGGGTTTCTAATGTTTTCTAGTGTTTTTAGT<br>GTCCTCGGGGGAAAAA

P. boreale putative satellites (low confidence)

Pol _B 1/14.0	2686	ACTAGAGGGTATTTTGGTCATTTCACTTGAAACGCTTTGCTTCCCTCGTCTC<br>TAGAATGAGTTTGGAGGGGAATGAACTCGGGGTTTTCAATACGAAAGTAT<br>TATAAGGCCATACTAGTGCTAGGGTTTGAAAATCAGAAAATTTGGAAAAT<br>CCTCCTAGCCCGGTATTGCCAAAGCTAAGTTACGAAGTAGGTTTCGGAATA<br>GTCAACGAATCCAAAGTTGACTTTTGAAAAGGCCAATATGATTTAAATCCG<br>AAAAGTGATTTTTGAAGACTAACTTTTGACACGATTCGAAACGTTTTGCAA<br>AATCTCATTTTAATGGCCTAACACTTAGCAAAATTTGCATTTTCTGGCATTT<br>CTGAAAACGGGGTATTTCAATCTTCCCCTCTAAAAACAAAGCTTCGTCCCC<br>GAAGCTGAACCATAGGCAACTCGGAAACGCAACTCTGAACACATAACACA<br>TAACATACGAGATGCATGTTCTAATGCAGATGAATGCCACATAAGACACC<br>AAGTAATCCAAAGGAGTCACGAGATAGTCAAACGACTACTCTGCTCAACG<br>AGGTTAAGACACCTCTGCTCAACGCAACCTATATCCTCGATATAGTGCTTC<br>CTACCAAGGTATCGCTAGCACAAGGCTACGGTCTCTCCGGTAAACAACGAT<br>TTTATAAAAGATGAATTTGAGTCGTGACAAACTTCAAATCGTATCGATGGT<br>TTAGAACTCATAAAACCGAGATTTTGATTTGCTACATTAATCGTTTCCAAA<br>ACTGATTATTAGGTCATTGACCAATCCCCTCAAGTTTCCCCACATAGGATA<br>GGAAAAACACGTAGCGTCGTCACACCCACGGAATCCTTCAGCTATCTCGAGT<br>GAGTGTCAGTGTTCCAACTAAGTATACCAGCGGGCACAACCGATAGCATC<br>GTCATACTATAGTCGCTCCAACGGACTGGCACACAAGTGGTTCACGTCACT<br>TTAGCATTTGGTACTCGAAAAATATGAGTAGAAATATTTTTACAAGTATAA<br>GTTTAAACTGTAGCGGTGAGAGTATGCAAAAATATGAATTTTACGATAATC<br>TCTGTTGTGTAGCTATCTCGAACGAATCGAAAGATGCTAAGCGCGTTCTAA<br>GGAAACGAAACTACTCTTACTCGACTACAAACAAGGTAACCAAGTAATAA<br>AGCGGAAAGTAATCACTTTGCACGCATCGATACAAATAACCATACAAGCA<br>TGTCAACATGTATTTCACATAAGCATGTCAATCATAACTCCATACCAACAT<br>GGCAGATTAGGCATCACAGCCAACTACAACATCTCACAAGGGAGAATCAA<br>GAACAAGCGCTAGAAGCGGCAATATCAATACCCAATCCCATCGGCTTATTC<br>AAGATTTTAACTTCTCAGTTTTAGTTGCTCCTAACTGGTTAAGCATTTTGAA<br>AAGGTTTTTCTTTACTTGTGATTAATCTTAGAGGTGGATAAGATTATAAAG<br>AGAACGCTGCTCTGATACCATCTGTAACACCCCGTAAATTCTAGGCCCTTC

		TCAAAAGACTCAGAAGATCCGGGTTGCCGACTCCGCAGCGGTGAAACACT ATCATGGAATGTGCCATGAATCGGTCTCACGTACTAAGAGTCTAAATGAGT GCGTTCAGTTCATAATCAAATCCAAACATTACCATATTAATCAAAAGACTT GATAGAGTCAAGCCAACGTTTTTATACAGAGTCTAAGCGGAAACTAAATA TTACAAAATATGAATCGCTAGCTATTACATATATGAATCATCAACTACGTC TACGATTACCGTCGGATCGTTTCTAAGTTGGTAGGACATGTAATACGTGAT CGATCCCATAACACCTGCAATCATTCTACGGTGGTTTGGAAAACAGACACC GCAGTAAAAGAGAGTTTAATCAGCAAAAGGCTGAGCAAGTAATATACCAC AGTATGTAAACTCGGAATGAAAACAGTTGATAAGTTGAAAACCTCCAATT CTGTTCATTTCCATTTCTCATGTGATACCAAGGGAATACTAATCCATTGTCA TCCAAAGGTCACCACTAACATCCTAAGGATATCAAGATTAACATCACAATC ATCACAGCACCAAGATATCCAAAGAACATCACCGATAACCTAAGGAAAAC AAGACAACACCCACTACAACATCATAAATCATCATCTAGCTCAAGAACACC AAGAACTACCATGGAAATCCATGGAAAAGCTCAAGAACATCAAGAACTAA CCATACAAACATCAAACTAACCATCAATAGCATGTCTACTACAACTATCAA CCATGAATCAAGCTTTAAATCAAAGAAAATCAAGTTTTGAAAATTTTGTCA AGATCACTAGATGGATCATAACTCAATCAAAACAAGTTGGAATTGAATTTT GAATGGAGATTGAGTGAGGGGGAGTACCTAGGGTTGTTGTGTAGCAAGGA GGGAGCAAGGAGATCAAGATTGCAAAAAGTTACTATTCACAGCAAGCTAT TTTACTATTCACAGCAGAATACTTTCTTCTTCTTTGAGAGAGATGAGAGAG AGTTCTTGTTTTGGTGAGTGTTTGATGTGTTGTGAGGGTGGTGATGAATAG GGTAAGTTATATAGTATTTTTTAACCTCCTCTTTGACTAATAAAAGATGAGT GGAAAGGAAGCTTCCTTGAAGCTTCACAAGAAAGATGAGGTTAAAGC
Pol _B 43/0.19	193	GAGTATTGTTTGGATTTCTAAATTTCTCATCCACACATTCAAGGGTTTACAC TGTAAATTAAAGAGGCGGGATAAACTATTCCACTTGTCATATTTTGGGGAT ACACTAATCCCCATAGTAGATTGTTATATAGATAGTTTGACTTTTGAATAG AAGTGGGACCAATATGGATTATTAGAAGTAAAAAAAAAA
Pol _B 51/0.14	177	TTTTTCAAAACTGTTTTATGGGTATTTAGTACACCTATGTATCATTGCTACT GTAAAGTTTCATGATTTTTCGACTTACGGTTTAAGAGATATTGAATTACAA AGTTGAAACCACAGATCAGAATTTCCTACTGGGCAGCTGTATGTTTTGGCC TATAACTCGTGAACCGTTCATCA
Pol _B 78/0.059	82	TTTGGTCAACTAGTAGTTCAATTGAGTACATTAGTACCGGTTGAGTGTCCG ATTGAGTGTTGAGTCCCAATTGATAGTCCAA
Pol _B 85/0.042	563	AATTGCTTATGAATTTGTCGGATATCATTTGTCAGATTATTACAGTTAAAG GTGATGAAATCGTCTACAAAGGACCATGTGTCAATATTTCAGGTTGCAACA ATTATTGAATTAAGAATGGTATACATGGCGAATATGTTTTCGTCCAGCAGT TATGAGTTTCGAGTGTTATTGTATTTTACAAAACTAAATGAACCGAATCGC TTCCAAAAACAAAAAAACACGTAACTAAAAATCAAAACTTCGCATCTATC CTAACTAAATTTATGGAGAGTATAAGTAGAAAATGTATGCAAGGAGCTTA ATACGAACTTGTTTACATGTTTGTTTTTGTCCCGAATTTAATTTATCTCATA TTTCCTATAAGAGACCAAATCAACACAAAATACTTCAGACAAAATGACTTC AAATAATCCACTCATATCACTAATGCTTTGTTCATAAGATTTCTCTCTGCAT CGCTTTGCTCTTAAGTTCAGGTTCAAACTTTATCTCTATTTTTATTAAGTGA AATATGTAATATTTGTGGTCGTTACAACAATTATTTGTGAATAATTTTTTTT
Pol _B 128/0.019	855	ATGTCCCCCTGGGAAATGCAAAGGAGCGCCAATCTAAACAACGCCACCCT TATCACGAGCGATAAGCACCGGCACCACAGCCCCCTACCACAGAAGCCCC TAAGGCTCTTAACAAACAATTCAAAGAAATCAGGGTGGAATATTAAGCAT AAAGACTTACCAAGAGAAAGATAATCAGTTACCTAAGAGAGCTCCAATGA CACAGCTAGATTTCTAGCCTGCAATCAAGAGAGAATTATCAATCCTATGAG TTAGAATCATATTGCAAATAACTTAAATACTATTATAGTAAATCTGCATCG GCTTTACAAAGAAATGATACTCAGGAAAGGCTCATTCACTACATTGGGACT TCAACAACAAAAGCAAGGTATAAATTGCAAACAACAAGTAATGAATTAGC GAGTACATTAAACCAACTATGGGGAACTTATCAAAATCAAGGAACCAAAA AGATACAATGCTCTACCAGAAATCACCCAACCCAAACCAATTCACAAATT AGGGTTGTTAACCACATTAATAGGGGTTGTTAAATTGAGAGAATAAAAAA CAAACTAAGAATATAAAGGGTTGTTACCTGTTGGAATCGCCCAGTGGAAA CTCAAACAGAGAAGAACCACTATCCGATTCAAAGTCTATCTTCAGCTGTGG CTGTATATCGTGGCATAAGTACCCTGCAAATATGAGGATTATAATGGTGAA

		TGAGATTGATAGCAGGCAATAGCAAACAATAAATCCCCCAATTTTAGGTTA<br>CTCCTCAGACCAAATAGTAAAAGGGTTGAGTTACAGATTAAATTATATGAT<br>GCCCTGAGTGAGAAAGAAAACAAAATGAGGGAGACTACCAAATAGAAG
Pol _B 165/0.012	97	TGAGGAGTTCCTAATACTTTTTAGACAAAATTGCCCTTATTAATCTTTAAC<br>CTTTTAATCACTATCTATACTCTTTATCTCTACTACTTAAAAGAA

P. villosissimum putative satellites (high confidence)

Pol _V 29/0.31	507	TGGGTTCACCACCACCACCTATCGATGTATCTCATTCACCATTATGATACA<br>CTAAACCTCAAGCATCAACAACAACATGATGTCTTTGGCCTAAGACATGGA<br>CTCTCATTTGGATTCCATATGTATTGAATGATCTAAGGGATTCACTCATTTG<br>TTTACATGTGCAAGGGATTCTCTTATGTCAACACTCTAGATGATTGTTATGG<br>TTTTCAGCCACTCAGGGTGAATGTTTTTCCATCTAATTTGGTAATTTTCATT<br>TTCTTTTGTAGTGTGCATGTTAGTGACAACGTCAAGGCACTCAATTAGAAA<br>AAAAAAACATAAAAGTAAATGTTTCTTCCAAAATGTTATAGTTTACCGCC<br>ATAATGTTTACACTTACCTCCACAACTTCACATAGAAATTGAAGCAACTTA<br>TGGCATTGTGCATTTAATTCTTTGCTACATGACATAAGTGGTTTTGAAACAT<br>ACCTTGTGACACTTACTTGTACAATAGTGAACATTTAATTCAAT
Pol _V 39/0.18	192	ACTTCTAATAATCCATATTGGTCCCACTTCTATTCAAAAGTCAAACTATCTA<br>TATAACAATCTACTATGGGGATTAGTGTATCCCCAAAATATGACAAGTGGA<br>ATAGTTTATCCCGCCTCTTTAATTTACAGTGTAAACCCTTGAATGTGTGGAT<br>GAGAAATTTAGAAATCCAAACAATACTCTTTTTTTT
Pol _V 146/0.012	544	CCTTTTGGATGATGAACAAGTCCTCTAACGGGCGTATTCTCTATTCTCCAG<br>ATTCTAGACATCACAGAAGGGACATATCTTCGACCTTTTCCTTTGTTAGAA<br>GCAATATATCGTACGACACAGATTTTTCCAATAGAAACTTACTTATATTAT<br>TTCATTAGATAAATAACAAAGAGTACACCTATTTTTCTTGTTTGTGTCTTTA<br>CTGCGTTATACATGGAGTAATATTTGGTTGAAAGTAGTTAGTTTGGAGGG<br>TAATAAATTCATGACTAGATTCCTAACAAGATTGATGTCGTCCAATCCTTC<br>CACGTCAGAATACGATGACCAGTGCCAGAAACCTAACAAGACCTATTTTTC<br>AGCGTGTATTTGATAGTAGTTTTTACAAAAAACAAAGCCAATTTCCAAAGT<br>CTCTTTGCGCCTCTCTGGATCGCACAACTAAAACGGTCTTCACCTTCACTTC<br>ATTCTGATCGATAGGTGCAATATGCTTCTTCCTTTTTTCCTTCACACATCTT<br>GTTGGTAACTACGACTCTTACTGCGATTGTG

P. villosissimum putative satellites (low confidence)

Pol _V 1/16.0	3608	TACTGAACGCGTACTACTTAAGTAGATGGTGGCTACCTTGCCCTTGTGGTA<br>TTCGGTACAAATTAATAATAATTGGACATTTATGATTAGACTTTGAATAAG<br>TCGAGATTGATCGACGGTTCTCTGATGAGACCGATCGGGTTTGATGAATGG<br>TTTGGAATTGGTATTCCCTTGGTATCACATGAGAAATGGAAATGAACAGAA<br>TTGGAGTTTTTCAACTTATCAACTGCTTTCATTCCGAGTTTACATGCTGTGG<br>TATATTACTTGCTCAGCGTTTTTGCTGATTAAACTCTTTTACTGCGGTGTCT<br>GTTTTCCAAACCACCGTAGAATGATGCAGGTTTTGTGGGATCGCTCATGTT<br>ACTACAATCAAAACAAACTTAGAACGATCCGATTAGAAATCACGATTAGT<br>GGTAGTTGGTGATTCTTATGTTGTAATGTAATGATTCATATATGTAATAGAT<br>AGTGATTCGGTTTTGTAATATTTACTTCCGCTTAGACTCTGTATAAAAACGT<br>TGGCTTGACTCTATCAAGTCTTTTGATTAATATGGTAATGTTTGGATTTGAT<br>TATGAACTGAACGCACTCATTTAGACTCCTTGCATGTTGAGACCAATTCAT<br>GGCACATTCCATGATAGTGTTCACCGCTGCGGAGTCGGCAACCCGGATCTT<br>CTGAGTCTTTTGAGAAGGGCCCAGAATTTACGGGGTGTTACAGATGGTATC<br>AGAGCAGCGTTCTCTTTATAATCTTATCCACCTCTAAGATTAATCACAAGT<br>AAAGAAAAATCTTTTCAAAATTCTTAACCAGTTAGGAGCAACTAAAACTG<br>AGAAGTTAAAATCTGGAATAAGCCGACGGGATTGGGTATCGGTGTTATTG<br>CTTAAGTGCTTGTTCTTAATTTTCTCAAGTAGAATGTTGTGGTCGGCCGAGT<br>TGCCTAAACCACCATGCTTATATGGAATTTTGTGTTGATATGCTTATGTGAA<br>ATTGCGGTAGCTAGTGAGACTATGAGAGTTCTCATTATGCTTATTCCTTTTA

		GAATCGTGACGTTGGTTGATGACGTCCGATATTGTTGCGAGCTTGACTCTT<br>AGACGAGTGGGCCGTTATGTGCGTCATCTTGATGCTTATGTGAATTGGTGT<br>GGTTATTTGTATCGATGCGTGCAAAGTGCTTACTTTCCGCTTTATTACTTGT<br>TTACTTTATTGTAATCGAGTAAGAGTAGTTTCGTTTCCTTAGAACGCGCTTA<br>GCATCTTTCGATCGTTCGAGATAGCCACAGTTTAAACTTATACTTGTGAAA<br>ACATTCTATTTTTCATAATTAACTGTCGAAGTACCAAATGCTAAAGTGATG<br>TGGACCACTTTGTGTATCAGTCCGTTGGCGCGACTTATAGTATGGCCATAC<br>TAACAGTTGTACCCGCTGGTATACTTAGTTGGAAACACTGAAACTCAGACG<br>AGATAGCCGAAGAATTTCGTGGTGTGGCGACACTACGTATTTTTCCTCTCC<br>GATGTGTGGAACACTTGAGGGGATTGGTCAGTGACCTAATAATCAGTTTTG<br>GAAACGACTAATGTAGCAAATCAAATTTCTAAATCATCGATACGATTTGAA<br>TTTTGTCACGACTCAAATTCATCTTTTATAAAATCATTGGTTACCTGAAGAG<br>GACCCGGAAGAGACCATAGCCTTTGTGCTAGCGATACCTTGGTAGGAAGC<br>ACTATATCGAGGATATAGGTTGCGTTGAGCAGAGGTGTCTTAACCTCGTTG<br>AGCAGAGTAGTCGTTTGACTATCTCGTAACTTCTTTGAATTACTTGGTGTCT<br>TATGTGGCATTCATTTGCATTAGAAGCACATGCATATTGTATCTTATGTGTT<br>ATGTGTTTAGAGTTGCGCCTCCGAGTTGCCGTGGTTCAGCTTCGGGGACGA<br>AGCTTTGTTTTAAGAGGGGAAGATTGAAATACCCCGTTTTCAGAAATGCAA<br>GAAAATGCAAATTTGGCTAAGTGTTGGGTCATTTAAATGAGATTTTGCAAA<br>ACGTTTCGGAACTTGTCAATCTTAGACTTCAAAAATGAGATTTCGAAAATA<br>AATCACTTTGGTCTTTTCAAAAGTCAACTTTGGATTCGTTGACTATTCCGAA<br>ACCTACTTCGTAACTTAGCTTTGGCAATCCCGGGCTAGGAGGATTATCCAA<br>ATTTCTTGATTTTCAAACCCCAGCACTAGTATGGCCTTATAATACTTTCCTA<br>GTGAAAACCCCAAGCTACTTCCCATCCAAACCCATTCAAGAGACGAGAAA<br>ACGAGTATCGACAACCAAATGACCAAAATGCCCTTGGTACCTTTTGACCTC<br>ATCACCTCAAAAGCTTACATCACTCTTGCCTATAAATACCCCTCTCTCTCAT<br>CACTCATACCCTTTGGTCAACACACAAAACACTAGCAAAATATCCCCAAAC<br>ACCATCCTCTCTCTCTCCACTCTCTCTCCTACCCAAAGCAAGACCAAGAGA<br>CACCAAAAACTGGTGCTCTCGGCCACATCAAGGAAGAATAACAAATTTTCT<br>TCAATCCTTGCCTCCTTGCTCCCTCCTTGCTACCAATCAATCCTTGGTAAGC<br>CCCCTCACTCAATCTCCATTCAAAGTTCAATTACAATTTGTTTTGATTTAGT<br>TATGATTTATCTTGTGATCTTGAGAAATTTTAAAAAGCTTGATTTTCTTTGT<br>TTTAAGGTTTGATTCATGGTTGTTAGAGGTAGTAGACTTGCTTTTGATGGTT<br>AGTTTGATGTTTGTATGGTTAGTTCTTGATGTTCTTGAGTAGTATGAGTTTA<br>GATGTTTGTTTTTATGTATTAGATGTTGTTAGCATGTTTTAGGGTTTTAGAA<br>AATGATTTGGATGTTAAGTGTTAATCTTGTAGATTAGTGGACTAATCAAGA<br>TTAGTGAATTGGTTTTATAAACTAAAACTAATCAACTTTGAAATTTGTGCCT<br>AAAACCTAAAACGAATCGCCTTGGGAAGCGTGCCCGAAAAGTCAAAGTCA<br>ACCAAAATTATTTTATTTTTCAAATAATTTTTAAATAATATTATAAGTCCAA<br>AATAATTATTTTGATAAACAAAGACGTTGTTTAGAATAAAAGTTAAACGTT<br>TTGTTCAAACTTGTTTTAAACTAACTTAGTCTTTAATTGAGTTATTATGTGA<br>TTAGGTTAGAGTGTGTTGTCTTTTAGTTTCGTTGAGCCGGGGTTAGTTCCCG<br>AGCGCTTTATGTGAACGTCTCGATAGTCGTGCCCCGTATGACTTATCGGAT<br>GTTAGTTTAGTAGTAGTCGAGTCTCACGTGCATTTAGGACATATCATTGTG<br>TGGTGTGCTTAGGATAATTACTAATGGTAATTATCATTAATTGTAGCCGAG<br>CGCTTCTTCGACGAAGGTTATTTCGACGCGGCGGCTTGAGTTCTTATTAGTC<br>TTCGCCTCTTCGGTTATACGGTGAGTTTTATATGCCAGTCTTTATTGCTTAA<br>ACTTCCAACGTGTTAAATGCTATTTGAAATGCGTGGATGTTTCTGCTTATGA<br>TTATTCAACTGTTTAAATGCTTTATGGATTAGTTTATTCAAAAATGTTATTT<br>AAACCTCGATTTCCAAAAGTCACGGTTCTACTATCTGAATGAACTAATACG<br>TAAACTTGAACGGT
Pol _V 3/7.3	2368	CGACCTTCTTAACCTATTTTCGACCTGGAGACGAACAGAACACAGCAGAG<br>AGGCAGTTTGGAGGTAGAGAGGAGCGTTCTTGGAGCAAGGAAGAGGACAC<br>AAGCGTGAAGATTGAAGATTCGAAGACGTGGATGCTCGGTTTGAGTCATC<br>CCTTCTCCTTTTCTTCTAGTATCTTCATGTCTTCTTCTAATCTATCTTGTAAC<br>TCTAAACTCATGAGTAGCTAATTTGTTTATCTATTGGGACCTTCATGGAATA<br>GCTAGGATTTGTGCCCAAACGACTTGATTTATATTTGATTCGTGCCTAGAA<br>CTCATTGATGGATTAATTTACACTATCGATTTTGTTTGAATGAATGAATTCG<br>CGACTTATTTGTTTTTACATGTTTATATTTGTGAATTGATCTATCTTTGAATA<br>GGGCTTAATGGATTTTGCGGAGTAGTTGGTATATACATATGTGAGTTTGAT<br>TAACCGGTGAGACCTAACGGAGATCTTGGTTAAAAGGATAACTGTGCGTA<br>TACCTAGGCGTATCCTGAGCCATAACAGGGATGGACTACCTGGGGGTTTGC

		CAGGGATATATGACTTATAGCTAGTTGTATACCTAGGGTAGACTTTATCTT<br>AATGCGTGCGTAGTTTGGTGCCCTAACAGGACCGTATAGGATTATGCATAG<br>AGACCTCTTAGTGCCGACCAAGCCTGAGGCAATTAAGTGCCTTACCTATCA<br>ATAGTACTCCAAATCCGCGTTTCTCAATGAATTAAAGTCAAGGCATGAAAA<br>CTAGCAAACCCCATGAAGTGTCCCAATAGTCCCTTCTCCATTATCACAAAA<br>ACCAAAACATTTTCTTGTTTTATTTTGCATGTTTATTTTCTTAGTATAGAAA<br>AACCAAACAATTTGCCCACATAAGTTAATTGACAACTAACAATCGCTCATG<br>GTCTTCGTGGGATCGATATAACTCCGGAATTTACTTACCGGTAAAACTACG<br>TCGACTACCGTGCACTTGCGGGAATCGATCAAGTTTTTGGCGCCGTTGCCG<br>GGGACTTCGGTTTAAATTAGTTGTAATTAGTAGTTTTAGGGATTTTTAGTTT<br>TTTTTTTCTTTTTTTTTTTTTTTTTTTTTTTTTTTAAAAAAAAAAAAAAAAAA<br>AAAAAAAAAGAAAAAGAAAAAAATATGGGGCTTTTCATTTTATTTATAGG<br>CCGAGTGGTATAGCTTTTTTATCCGTTGTGTGCAATAAGCTTCTCTCCATAT<br>TTCACTTTGATGACCACGGGATTCACCAGGGCTTGAGGGACGCCGAGGGT<br>GTTGGTGAGATTGTTGGTATTCCATCTTAGTGAGGGTGTGTGAGATAATTC<br>GGGTATGGTGAGGCAAATGACGCATAGGAAGTATTGAGCTACATATATAT<br>ATCTATCCTACCGTCCTTTAGCCTTCATCATAAGACCTTTAGCGGATTAGGA<br>GCTCGGGCCATCTTCGTGGCTAAGCCCGTAGTAAACTCTACCTTTTAGCAC<br>ACATGAAAGGGGAGGTATTTTTAGAGAGTAGGGTTAAGTTCTTTATACTCA<br>TTAGGGGGATGTCGTGTTAGTCAAAATTTGGTTTCTTGTCACGCTCCTTTCC<br>TACGAGTCTTATAACTCACGAAGTAACAAAGGATTCTAATGAGCTTGCCCG<br>CTCTCACAAAAAGAAAAATAAAAATTTTTTTGCATGCATTTAGTTTAGGTA<br>TTTTGCATAGTCATAGTCCTTGCATTGCATGTAGTTTTGTTTTAATCGAGTC<br>TCGTTTTGTGTCGTGACATGGTTGACATTGGTGTCGAGAGTCGGCATGATA<br>CATGGGGGATTCGAGGCTAGCATGCATGTTGAGAGAGTGTGGTCGCTCGG<br>GGCGAGCTTTAGGTAAGTGTGGGGGTATTTGATAGCACTAGAATTAGTGCT<br>ATATTTCATGCCATTTATCATTAGCTTTGCCTCGATTTGGTGCGATATATGA<br>TGAGATTCCTATGATTTAAGCTTGTATTCCGCTTTGTGACTCCACGGACGTG<br>ATTACCACTCTGTTGCATGATTTCGAGCTCATTTGTCCCTTATTTTGTCATA<br>TATGATCGCGTGGTAGTTTGTTGCTCGTTTTGTGCAGGAAACAGGATCGCG<br>AGGCTATCATTTGGACGAGCTCGGAACACCGGAGATGAGGAGGAACATGG<br>TGGAAGAAAGATGATCAAGGATGCAAAGGAAAAAGACCCCAAAAAGACA<br>TGCCAAAGACACCACAAGCTGAGGAAAGCAAAGGAAAAGGTCACTGCCA<br>GTGACCGTTCGCGCGAACGCCCCAAGCGTTCGCACGAACGGCGTTCGCAC<br>GAGCGTTCGTGCGAACGCTGTCGGGGGCCAGAATCGGAATTTCGACCTTTC<br>ACTTGGAATTACGGGATTT
Pol _V 33/0.22	177	ACACCTATGTATCATTGCTACTGTAAAGTTTCATGATTTTTCGACTTACGGT<br>TTAAGAGATATTGAATTACAAAGTTGAAACCACAGATCAGAATTTCCTACT<br>GGGCAGCTGTATGTTTTGGCCTATAACTCGTGAACCGTTCATCATTTTTCAA<br>AACTGTTTTATGGGTATTTAGT
Pol _V 59/0.068	37	CACTAAAAACACTAGAAAACATTAGAAACCCTAAGGA
Pol _V 66/0.046	89	TAAACATGTATTTTTGAAAATAAAAATGATAAATATCTTACTAATGGGTCA<br>TTAATTTCAATGGGCCTCTTTTGAGCCCAAACAAAAAA
Pol _V 130/0.015	512	GTATTAAATTAAAAAAATTAAGATGTACTCTTCAAAAATCTTTCGCTGCAA<br>AGCTGCATACAAACAACATAAAAATTCTGGTCTACCCAATCCTACAACTCT<br>TAACAATATTAAACAGGATCATGTGAGATCAGAGCAATGAGAAGAATTTT<br>TGCTTTAACCCCTTAAACTGAACATTTAACAATAATCTTTTTAATCAGAGTC<br>TAATCTTAGACTCGTTTTCAATCCAAATACACAAAAGAAGATTGATTCTTG<br>TTAATAAAAATGAAACACTCCAATTACAAAATTATAATAAAGATGTTTATT<br>GAATAAAAAATTAAACAACATGAAATCTAAAGCAAAAAAGAGAAGATAC<br>CTTGTGGATAAATTGACTTTTGGTTAATCATCCACTTCAAAGCTGCATACG<br>AAGAAGAAGAGGAAGGAGTCTTTCACATAGCTTGGATAATAGCTGAAATG<br>GTGGTGATTATCATAAGTTGTGAGTAAGTGGCCATCTAAGTGGGTTCATTT<br>GGTTA
Pol _V 131/0.015	168	ACTTGGTGATCGACTGAGACTATCGGTTCAATTTGACTAGTAACCATAGTT<br>TGGGGATAAATACCGAGATAGACCAATTAAGTCAATTGGAGTTGTAAGTG<br>GTACTAATTAATCATAGCTAATTACCATATGTACCAATTTCAAGTAACCGG

		TGTGACCTCTCGGGTC
Pol _V 144/0.012	563	TGTTTTTAGAAACAATTCGGTTCATTTAGTTTTGGAAAATACAATAACACTCGAAACTCATAACAGGTAGACGAAAACATATTCGCCATGTATACCATTCTTAATTAAATAATTGTTGCAACCTGAAATATTGACACATGGTCCTTTGTAGACGATTTCATCACCTTTAACTGTAATAATCTGACAAATGATATCTGACAAATTCATAAGCAATTAAAAAAAATTATTCACAAATAATTGTTGTAACGACCACAAATATTACATATTTCACTTAATAAAAATAGAGATAAAGTTTGAACCTGAACTTAAGAGCAAAGCGATGTAGAGAGAAATCTTATGAACAAAGCATTAGTGATATGAGTGGATTATTTGAAGTCATTTTGTCTGAAGTATTTTGTGTTGATTCGGTCAATTATAGGAAATATGAGATAAATCAAATTCGGGGCAAAAATAAACATGTAAACAAGTTCGTATTAAGCTCCTTGCATACATTTTGTACTTATACTCTCCATAATTTTAGTTGGGATAGATGCGAAGTTTGATTTTTAGTTACGTGCTTTTT

Figure S1. FISH-based localization of 5S rDNA (red), 45S rDNA (green) on the metaphase spreads of *P. pulcherrimum* (A), and *P. racemosum* (B). Localization of satDNAs Pol_C 33 (green) and Pol_C 46 (red) on the metaphase spread of *P. kiushianum* (C) and DAPI invert image of chromosomes (E). Arrowheads indicate the heteromorphism of chromosome 7 homologs in signal size of 5S rDNA in *P. pulcherrimum* (A) and the reduced number of 45S rDNA clusters (5) observed on chromosomes of *P. racemosum* (B). Arrows point to Pol_C 33 and Pol_C 46 localized in the DAPI-positive bands on chromosomes of *P. kiushianum* (C,E). The correspondent probes and their pseudocolors are specified on the left. Bar – 5 μm.

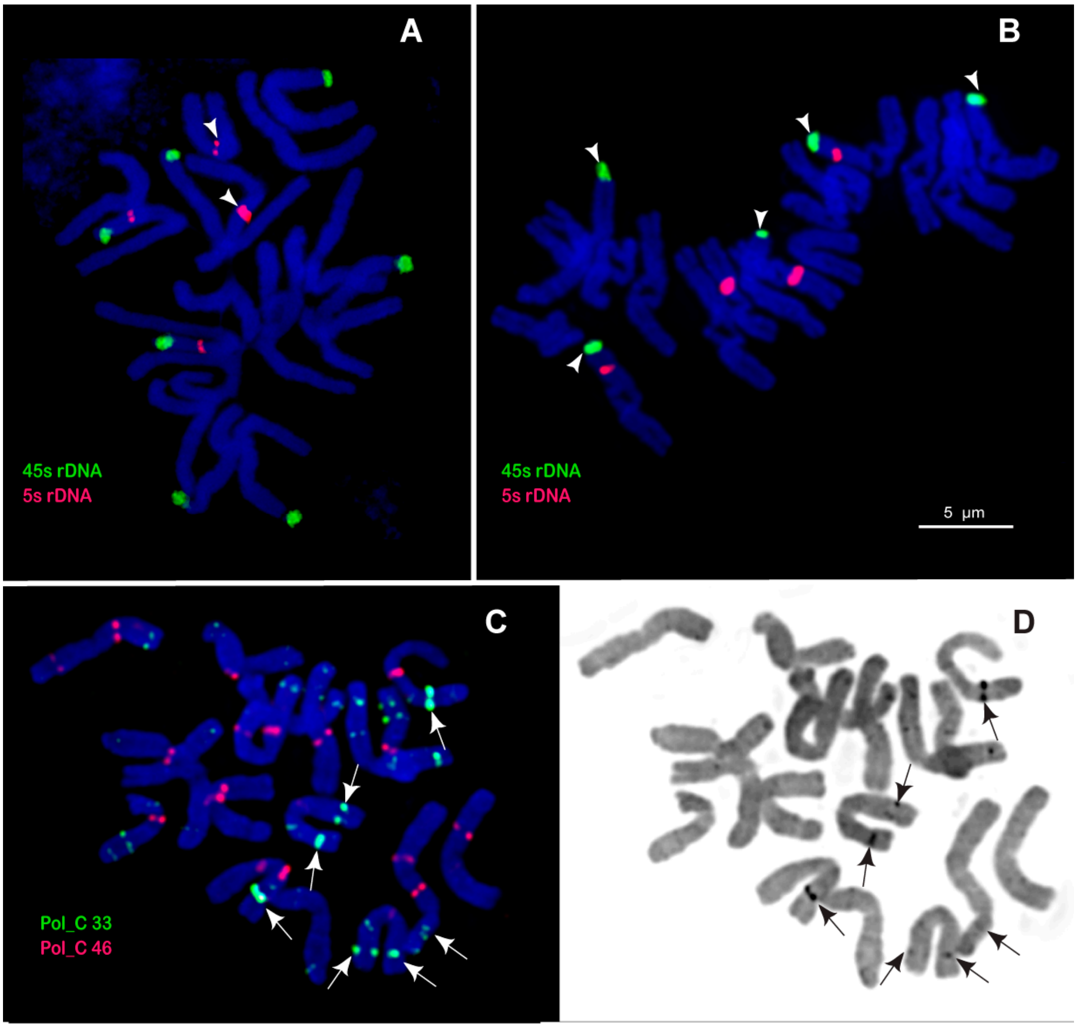

Table S3. List of the oligonucleotide FISH probes.

Oligo FISH probe name		Oligo FISH probe sequence
Pol_C 33	Pol_C 33_1	CTCATTTGCATTCCATAGGCATTGTATGATCTAAGGGATTCACTCCTA
	Pol_C 33_2	AAATGTGTTTCCATCTAATTTGGTGGCCAACATGTTGGTGA
Pol_C 46	Pol_C 46	TGGGGATTAGTGTATCCCCAAAATATGACAAGT GGAATAGTTT ATCCCGCCTCTTTAATTT
